# In Type 2 Diabetes Mellitus, normalization of hemoglobin A1c accompanies reduced sensitivity to pressure at the sternum

**DOI:** 10.3389/fnins.2023.1067098

**Published:** 2023-06-14

**Authors:** Jens Faber, Søren Ballegaard, Nanna Ørsted, Ebbe Eldrup, Benny Karpatschof, Finn Gyntelberg, Sofie Korsgaard Hecquet, Albert Gjedde

**Affiliations:** ^1^Department of Endocrinology, Herlev-Gentofte University Hospital, Herlev, Denmark; ^2^Faculty of Health and Medical Sciences, University of Copenhagen, Copenhagen, Denmark; ^3^Department of Psychology, University of Copenhagen, Copenhagen, Denmark; ^4^The National Research Center for the Working Environment, Copenhagen, Denmark; ^5^Department of Neuroscience, University of Copenhagen, Copenhagen, Denmark; ^6^Translational Neuropsychiatry Unit, Aarhus University, Aarhus, Denmark

**Keywords:** glucose homeostasis, homeostasis, autonomic nervous system dysfunction, type 2 diabetes, pressure pain sensitivity, glycated hemoglobin (HbA1c), energy homoeostasis, glucose metabolism

## Abstract

**Background:**

The autonomic nervous system (ANS) maintains glucose homeostasis. While higher than normal glucose levels stimulate the ANS toward reduction, previous findings suggest an association between sensitivity to, or pain from, pressure at the chest bone (pressure or pain sensitivity, PPS) and activity of the ANS. A recent randomized controlled trial (RCT) of type 2 diabetes (T2DM) suggested that addition of an experimental, non-pharmacological intervention more effectively than conventional treatment lowered the levels of both PPS and HbA1c.

**Materials and analyses:**

We tested the null hypothesis that conventional treatment (*n* = 60) would reveal no association between baseline HbA1c and normalization of HbA1c in 6 months, related to change of PPS. We compared the changes of HbA1c in PPS reverters who experienced a minimum reduction of 15 units of PPS and in PPS non-reverters who experienced no reduction. Depending on the result, we tested the association in a second group of participants with addition of the experimental program (*n* = 52).

**Results:**

In the conventional group, PPS reverters experienced normalization of HbA1c that corrected the basal increase, thus disproving the null hypothesis. With the addition of the experimental program, PPS reverters experienced similar reduction. The reduction of HbA1c among reverters averaged 0.62 mmol/mol per mmol/mol increase of baseline HbA1c (*P* < 0.0001 compared to non-reverters). For baseline HbA1c ≥ 64 mmol/mol, reverters averaged 22% reduction of HbA1c (*P* < 0.01).

**Conclusion:**

In consecutive analyses of two different populations of individuals with T2DM, we demonstrated that the higher the baseline HbA1c, the greater the reduction of HbA1c but only in individuals with a concomitant reduction of sensitivity to PPS, suggesting a homeostatic effect of the autonomic nervous system on glucose metabolism. As such, ANS function, measured as PPS, is an objective measure of HbA1c homeostasis. This observation may be of great clinical importance.

## Introduction

Living organisms modulate organ functions in adaptive responses to the environment. Processes of homeostasis maintain constant conditions of the internal environment by balancing opposing sympathetic and parasympathetic processes, as directed by the autonomic nervous system (ANS) (Cannon, 1926; Goldstein, 2019). We do not fully understand the specific neurophysiological bases of different targets of homeostasis, but we consider the processes generally essential to survival (Goldstein, 2019). Chronic diseases such as Type 2 Diabetes Mellitus (T2DM) typically relate to disruption of homeostasis at one or more steps of the biochemistry of organ functions (Goldstein, 2019). Levels of hemoglobin A1c (HbA1c) relate to the adequacy of the autonomic processes that regulate blood glucose concentrations and hence to the energy metabolism of the body, in part or in whole as supervised by orexin receptors in the lateral hypothalamus (Tsuneki et al., 2012; Adeghate et al., 2020). We assume that the homeostatic normalization of blood glucose is more active with increasing levels of blood glucose. Thus, with higher levels of HbA1c at baseline, we expect mechanisms of normalization of HbA1c to intensify because of the influence from ANS.

It remains unknown to which extent the phenomenon of elevated sensitivity of pain to pressure at the chest bone called pressure or pain sensitivity, PPS (Ballegaard et al., 2014)] may be a general sign of disrupted homeostasis associated with elevated sympathetic tonus of the ANS. In a previous study, Faber et al. (2021) tested the specific claim that the PPS of the chest bone periosteum may reflect the degree of disruption of the ANS. In a randomized controlled trial of 144 individuals with T2DM, the authors questioned whether 6 months of a specific supplemental non-pharmacological treatment would reduce elevated PPS measures and improve peripheral glucose metabolism. The authors observed that PPS and HbA1c measures underwent a correlated decline and concluded that the proposed supplement to pharmacological treatment of T2DM could be an effective addition to conventional therapy (Faber et al., 2021).

There is evidence from the literature that stimulation of body surfaces or peripheral nerves has specific effects that may arise from involvement of the central nervous system. The methods include vagal and sacral nerve stimulations (Kupers et al., 2006; Lundby et al., 2011; Landau et al., 2015), and spinal cord stimulation (Tesfaye et al., 2010; Richner et al., 2019). Previously, Ballegaard et al. (2014) observed that non-pharmacological reduction of elevated PPS measures in healthy volunteers accompanied reductions of serum cholesterol, blood pressure, heart rate, and work of the heart determined as the pressure-rate product. The authors also reported correlation between reductions of PPS and HbA1c values in healthy volunteers, suggesting that reduction of PPS relates to regulation of blood glucose (Ballegaard et al., 2014). Whether reduction of HbA1c in individuals with T2DM has direct links to reduction of PPS at the chest bone nonetheless remained an unconfirmed conjecture.

As the test of the conjecture, the present study used previously unreported findings from the RCT noted above to compare changes of HbA1c over 6 months to baseline HbA1c levels, in PPS reverters who experienced a reduction of elevated PPS measures, and in PPS non-reverters who obtained no such reduction. We tested the null hypothesis that conventional treatment would not reveal an association between baseline HbA1c and the change of HbA1c in 6 months related to changes of PPS, in individuals with T2DM undergoing the conventional anti-diabetic therapy reported by Faber et al. (2021). Upon rejection or confirmation of the null hypothesis, we tested the revised hypothesis of a relation to change of PPS in a separate group of individuals who received an experimental, non-pharmacological therapy in addition to the conventional treatment.

## Methods

We completed a novel analysis of new findings of values of HbA1c recorded from individuals originally recruited and tested by Faber et al. (2021). We compared HbA1c values of PPS reverters and non-reverters in two separate groups of individuals with T2DM. We defined PPS reverters as individuals who obtained a decline of elevated PPS measures of at least 15 arbitrary units (a.u.) during a period of observation of 6 months, previously defined as the minimum significant reduction of PPS that represented 50% of an elevation of at least 60 a.u. We defined PPS non-reverters as individuals who did not experience this reduction. The analysis included two groups totaling 112 participants of the RCT who completed the study period of 6 months. Of these, 60 participants received conventional anti-diabetic therapy, and 52 received the experimental non-pharmacological treatment in addition to the conventional therapy (Faber et al., 2021).

### Design

General practitioners recruited eligible participants with T2DM, defined as individuals with baseline values of HbA1c ≤ 75 mmol/mol and PPS values ≥ 60 a.u. The criteria excluded patients with values of HbA1c > 75 mmol/mol that required referral to a specialized diabetes unit. Of 192 individuals screened, we randomized 144 participants of whom 112 completed the 6 months experimental period, described in detail by Faber et al. (2021). We used the magnitude of HbA1c reduction in relation to baseline HbA1c measures as the primary endpoint of individuals experiencing the predefined minimum reduction of 15 PPS a.u. of a predefined minimum elevation of 60 PPS a.u. during 6 months of observation. The participants received standard care according to national guidelines for the treatment of T2DM, including medical counseling, medical standardization, and education in lifestyle adjustments, and with their anti-diabetes medication being unchanged during the last 3 months prior to inclusion. We divided the 112 participants into two experimental groups. Of these, we told members of the conventional intervention group that PPS measures were elevated, possibly as reflection of a physiological strain on the body from the disease, and potentially with a negative impact of the prognosis of the disease. They received no further information or guidance. We assigned the members of the experimental intervention group to a non-pharmacological self-care stress management program (UllCare^®^) described in detail previously (Faber et al., 2021). The experimental intervention included daily PPS measurements at home for biofeedback and cognitive reflection, daily peripheral sensory nerve stimulation applied by finger pressure with the aim to reduce the elevated PPS measure, and continuous professional surveillance allowing pro-active contact in cases of deviation or missing measures. In patients with ischemic heart disease, we previously found that PPS measures matched assessments from questionnaires for depression, quality of life, self-reported health, and a broad range of clinical signs of ANS dysfunction, including physical, mental, emotional, and social dysfunctions (Bergmann et al., 2013). The participants in the present study answered the questionnaires on the day of PPS measurement to ensure no between-group differences or time effects.

### Hypothesis generation

We conducted the hypothesis-generating experiment as a test of the null hypothesis in 60 individuals randomized to conventional treatment of passive observation. The null hypothesis claimed that conventional treatment alone would not reveal an association between changes of HbA1c and baseline HbA1c values after 6 months of observation, in relation to reduction of PPS in individuals with T2DM undergoing conventional anti-diabetic therapy. We grouped the participants into PPS reverters and PPS non-reverters according to the PPS reduction.

### Hypothesis testing

Depending on the rejection or confirmation of the null hypothesis in the first analysis, we tested the resulting hypothesis in a second group representing 52 randomly selected subjects who underwent additional non-pharmacological 6-month intervention. The intervention again aimed at a minimum reduction of 15 a.u. of the minimally elevated PPS values of 60 a.u. or above, and we again grouped the participants into PPS reverters and PPS non-reverters according to the PPS reduction.

### Stratification of clinical effect

We stratified the results into three predefined groups of individuals according to baseline values of HbA1c. The groups included well-regulated subjects of group A with HbA1c < 53 mmol/mol, moderately regulated subjects of group B with 53 mmol/mol ≤ HbA1c < 64 mmol/mol, and poorly regulated subjects of group C with HbA1c ≥ 64 mmol/mol, based on limits used in clinical practice as defined by Hodgson et al. (2022) and Nathan et al. (2013). We evaluated the effect in each of the three groups by recording numbers of PPS reverters and non-reverters in the conventional and conventional-plus-experimental treatment groups.

### Statistics

We applied multivariate regression with change of HbA1c from baseline to end of treatment as dependent variable and baseline HbA1c as independent variable for participants randomized to conventional intervention or conventional-plus-experimental non-pharmacological intervention. We presented the results as the difference between the linear regressions of change of HbA1c vs. baseline HbA1c of PPS reverters and non-reverters. We subsequently divided baseline HbA1c into three categories and performed two-way ANOVA with the same dependent variable. We completed model validation by inspecting plots of standardized residuals against fitted values and independent variables and QQ-plots of the standardized residuals. We performed the statistical analyses in R version 3.60.

The distinction between the active conventional-plus-experimental and the passive conventional treatment groups allowed for adjustment of regression toward the mean represented by the PPS non-reverters after conventional intervention. The slopes of change of HbA1c values during the intervention plotted vs. baseline HbA1c values provided an index of the degree of normalization of HbA1c values. The normalization of elevated HbA1c measures in the three groups of incrementally increased baseline HbA1c values enabled the evaluation of clinical relevance of the observed effects.

We used the Cohen's effect size as a supplementary assessment of the clinical effect. We calculated the Cohen effect sizes as the differences of mean values of pre- and post-treatment measures of the active conventional-plus-experimental and passive conventional treatment group members, divided by the pooled standard deviation (Hedges and Olkin, 1985; Bech, 2012). In relation to clinical significance, a Cohen effect size of < 0.2 represents a minor clinical effect, 0.2–0.4 a small effect, 0.4–0.7 a moderate effect, and ≥0.7 a large effect (Baer, 2010).

## Results

At baseline, we found no significant between-group differences with respect to demographic factors, medication, medical history, physiological and biochemical health risk factors, and the questionnaire-evaluated self-reported health, mood, quality of life, and clinical signs of ANS dysfunction (Table 1).

**Table 1:** Baseline characteristics of randomized participants.

**General information**	**Conventional-plus-experimental**	**Conventional**
Number of participants	52	60
Age (years); mean (range)	63.8 (43–75)	65.9 (45–77)
Sex: male/female (number)	32/20	40/20
Diabetes duration (years)	10.3 (1–25)	9.9 (1–24)
Primary diabetes control unit: General Practitioner (%)	65	60
**Medication**		
Metformin [number of participants (%)	41 (79)	47(78.3)
Insulin [number of participants (%)]	6 (12)	10 (16.7)
GLP-1 agonist [number of participants (%)]	6 (12)	11 (18.3)
SGLT-2 inhibitor [number of participants (%)]	5 (10)	9 (15.0)
DPP-4 inhibitor [number of participants (%)]	8 (15)	16 (26.7)
Sulfonylureas [number of participants (%)]	3 (6)	5 (8.3)
Statins [number of participants (%)]	37 (71)	41 (68.3)
ACE/ARB [number of participants (%)]	27 (52)	33 (55)
Diuretics [number of participants (%)]	18 (35)	14 (23.3)
Calcium channel blockers [number of participants (%)]	17 (33)	13 (21.7)
ASA [number of participants (%)]	15 (29)	17 (28.3)
**Medical history**		
Peripheral arterial disease [number of participants (%)]	2 (3.8)	1 (1.7)
Previous myocardial infarction [number of participants (%)]	4 (7.7)	2 (3.3)
Previous CABG or PCI [number of participants (%)]	4 (7.7)	1 (1.7)
Previous stroke [number of participants (%)]	2 (3.8)	0
Previous treated depression [number of participants (%)]	7 (13.5)	8 (13.3)
Asthma [number of participants (%)]	10 (19.2)	3 (5.0)
Previous cancer [number of participants (%)]	6 (11.5)	2 (3.3)
Symptomatic neuropathy [number of participants (%)]	6 (11.5)	9 (15.0)
PPS (arbitrary units) [mean (SD)]	76.6 (12.4)	77.5 (13.6)
**Biochemistry**		
Hemoglobin A1C (HbA1C) (mmol/mol) [mean (SD)]	53.8 (8.4)	53.8 (10.8)
Creatinine (μmol/l) [mean (SD)]	72.8 (14.4)	77.2 (21.9)
Cholesterol (mmol/l) [mean (SD)]	4.1 (0.99)	4.01 (0.96)
LDL-cholesterol (mmol/l) [mean (SD)]	2.0 (0.78)	2.01 (0.8)
HDL-cholesterol (mmol/l) [mean (SD)]	1.27 (0.41)	1.27 (0.35)
Triglyceride (mmol/l) [mean (SD)]	2.19 (1.06)	1.77 (0.87)
**Physiology**		
Systolic BP (mmHg) [mean (SD)]	136 (16)	135 (17)
Diastolic BP (mmHg) [mean (SD)]	80 (8)	79 (7)
Heart rate (beats/min) [mean (SD)]	71 (12)	68 (10)
BMI [weight(kg)/height] m^2^ [mean (SD)]	29.5 (4.2)	28.4 (4.4)
**Health questionnaires [adopted from Bergmann et al. (** **2013** **)]**		
Treatment satisfaction (DTSQ) [mean (SD)]	25.3 (6.9)	27.3 (6.7)
Empowerment (DES–SF) [mean (SD)]	30.6 (5.4)	30.5 (6.2)
Depression (MDI) [mean (SD)]	8.9 (9.6)	8.7 (11.7)
Clinical stress score (CSS) [mean (SD)]	10.8 (11.3)	10.2 (10.9)
Quality of life (WHO−5) [mean (SD)]	66.4 (11.3)	70.8 (18.1)
Mental composite score (SF−36) [mean (SD)]	48.1 (9.8)	51.0 (10.2)
Physical composite score (SF 36) [mean (SD)]	49.1 (8.0)	48.3 (8.5)

We observed no significant differences between Active (Conventional-plus-experimental) and passive (Conventional) group participants. GLP-1, Glucagon Like Peptide 1; SGLT2, Sodium Glucose Transporter 2; DPP-4, Dipeptidyl Peptidase 4; ACE/ARB, Angiotensin Converting Enzyme/Angiotensin Receptor Blocker 2; ASA, Acetyl Salicylate Acid; CABG, Coronary Artery By-Pass; PCI, Percutaneous Coronary Intervention; PPS, Pressure Pain Sensitivity; BP, Blood Pressure; BMI, Body Mass Index; DTSQ, Diabetes treatment satisfaction questionnaire; DES-SF, Diabetes empowerment questionnaire; MDI, Major Depressive Inventory; WHO-5, quality of life score; SF-36, self-reported physical and mental health.

### Hypothesis generation by conventional intervention

**Figure 1:**
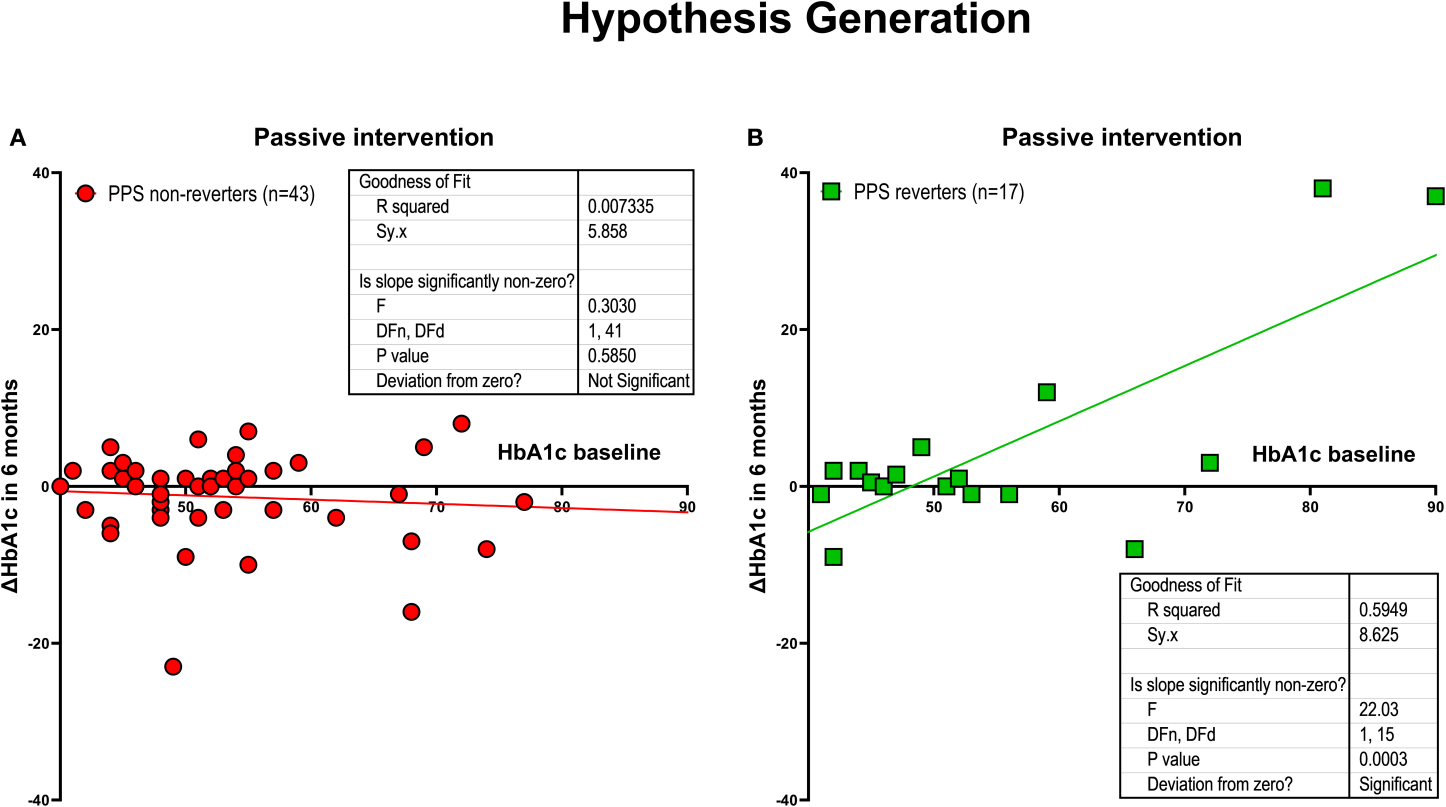
Hypothesis generation. Intervention effect measured as the relation between changes in HbA1c after 6 months of conventional treatment and the baseline HbA1c for individuals randomized to the conventional treatment group. The participants included individuals who experienced a predefined minimum reduction of PPS ≥ 15 after the intervention period (i.e., PPS reverters; *N* = 17) and individuals who did not experience this reduction (PPS non-reverters; *N* = 43). **(A)** PPS non-reverters; **(B)** PPS reverters (between-group difference significant at *P* < 0.0001). Regarding HbA1c change depicted on the y-axis, a positive value reflects a reduction in HbA1c.

The change of the HbA1c values during the 6-month period of observation in relation to the baseline measure of HbA1c in the conventional treatment group rejected the null hypothesis, as revealed by the changes of PPS reverters (*n* = 17) and PPS non-reverters (*n* = 43). The correlation between baseline HbA1c and change of HbA1c during the 6 months of intervention was r = −0.086 (*P* = 0.59) for the PPS non-reverters, and r = +0.77 (*P* = 0.0003) for the PPS reverters. The mean effect, measured as the degree of reduction of HbA1c averaged −0.71 mmol/mol per mmol/mol of increase of baseline HbA1c (95% confidence limits −0.49 to −0.92 mmol/mol, between-group significance *P* < 0.0001), as shown in Figures 1A, B.

### Hypothesis testing of conventional-plus-experimental intervention

**Figure 2:**
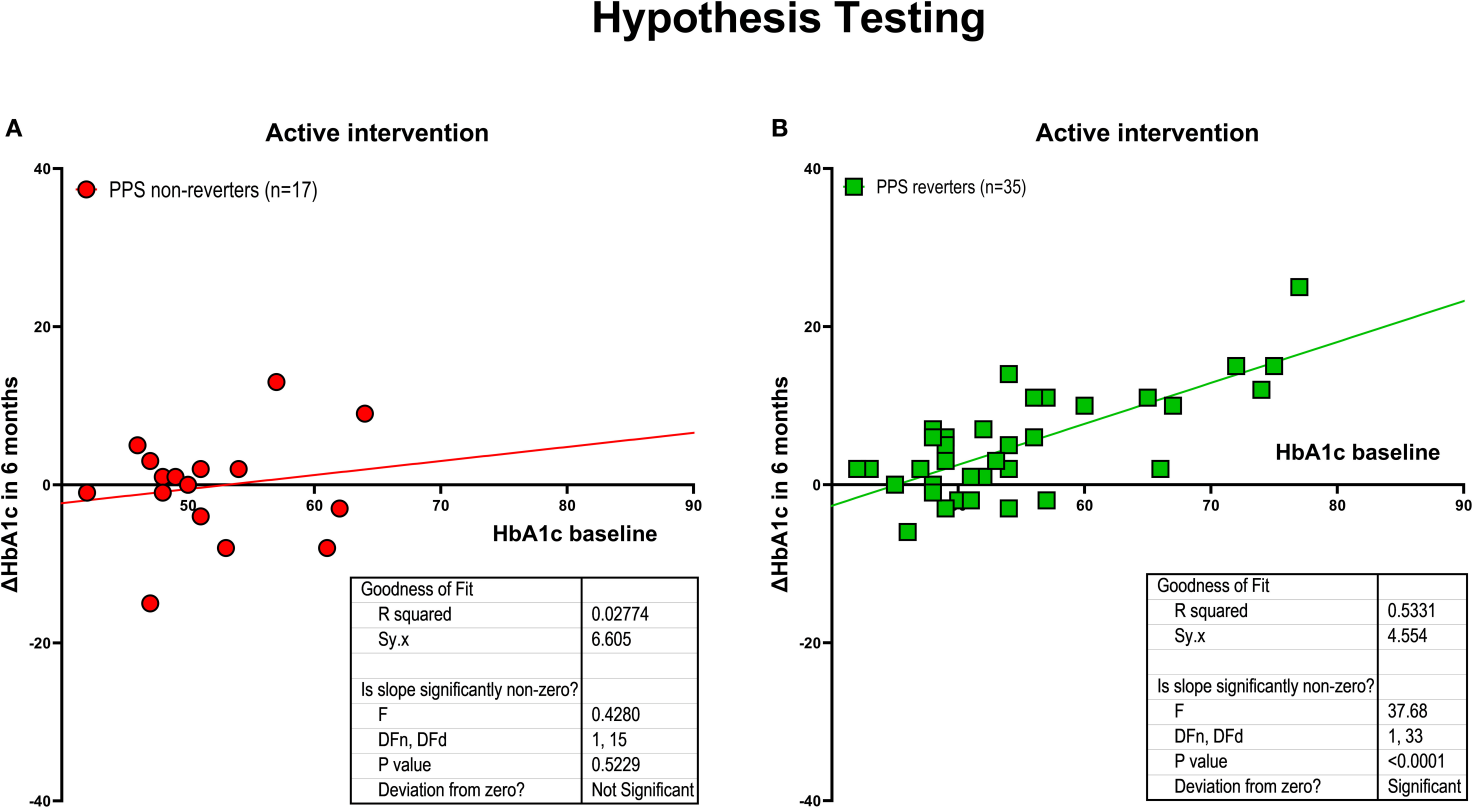
Hypothesis testing. Intervention effect measured as the relation between changes in HbA1c after 6 months of intervention and baseline HbA1c, for individuals randomized to active treatment group, including individuals who experienced a predefined minimum reduction of PPS ≥ 15 during the intervention period (i.e., PPS reverters; *N* = 35), and individuals without this reduction (PPS non-reverters; *N* = 17). **(A)** PPS non-reverters. **(B)** PPS reverters (between-group difference significant at *P* < 0.003). Regarding HbA1c change depicted on the y-axis, a positive value reflects a reduction in HbA1c.

The rejection of the null hypothesis led to the revised hypothesis tested in the group of conventional treatment supplemented by experimental intervention. In the experimental intervention group, we tested the revised claim that reduction of the HbA1c measure over a period of 6 months of conventional treatment supplemented by non-pharmacological intervention would relate significantly to baseline HbA1c measures only in PPS reverters. For the PPS non-reverters of the experimental group (*n* = 17), the correlation between baseline HbA1c values and change of HbA1c values remained insignificant, r = +0.17 (*P* = 0.52). In contrast, for the PPS reverters (*n* = 35), the correlation was highly significant, r = +0.73 (*P* < 0.0001). The average degree of reduction of HbA1c relative to baseline HbA1c values was −0.52 mmol/mol per mmol/mol increase in baseline HbA1c (95% confidence limits −0.29 to −0.74 mmol/mol with between group significance at *P* = 0.022) as shown in Figures 2A, B.

### Combined conventional and conventional-plus-experimental interventions

**Figure 3:**
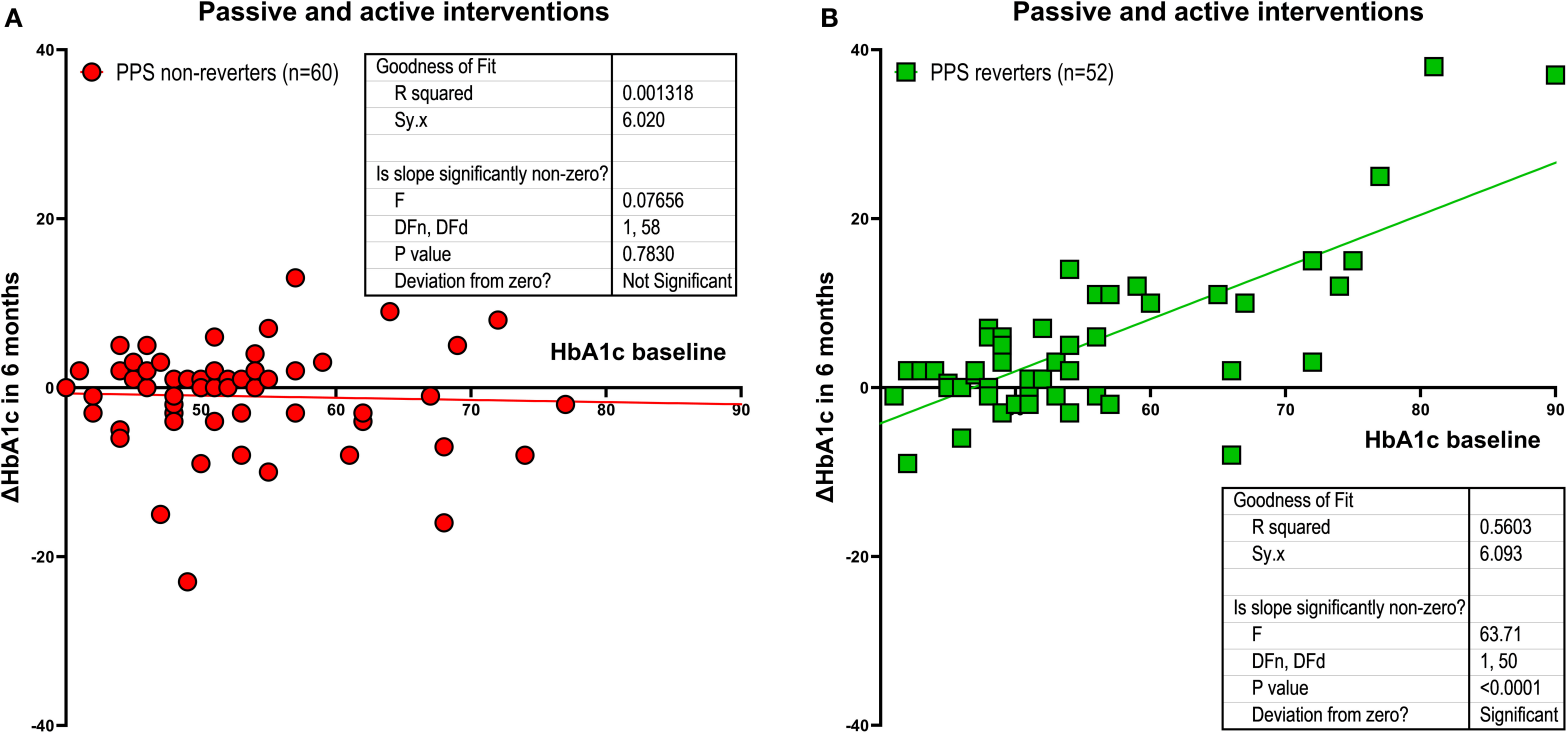
Combined groups. Intervention effect measured as the relation between changes in HbA1c after 6 months of intervention and baseline HbA1c with experimental and conventional treatment group members pooled, including individuals with a predefined minimum reduction of PPS ≥ 15 at the end of the intervention period (i.e., PPS reverters; *N* = 52), and individuals without this reduction (PPS non-reverters; *N* = 60). **(A)** shows PPS non-reverters. **(B)** shows PPS reverters (between-group difference signifcant at *P* < 0.0001). Regarding HbA1c change depicted on the y-axis, a positive value reflects a reduction in HbA1c.

For the combined groups of PPS non-reverters (*n* = 60), we found no correlation between baseline HbA1c and change in HbA1c during the 6 months of intervention with r = −0.04 (P = 0.78). In contrast, for the combined groups of PPS reverters (*n* = 52), the correlation coefficient reached r = +0.75 (*P* < 0.0001). The effect in PPS reverters, measured as the average reduction of HbA1c, averaged −0.62 mmol/mol for every mmol/mol of increase of baseline HbA1c (95% confidence limits −0.46 to −0.77 mmol/mol, between-group significance *P* < 0.0001) as shown in Figures 3A, B.

### Stratification by baseline HbA1c

**Figure 4:**
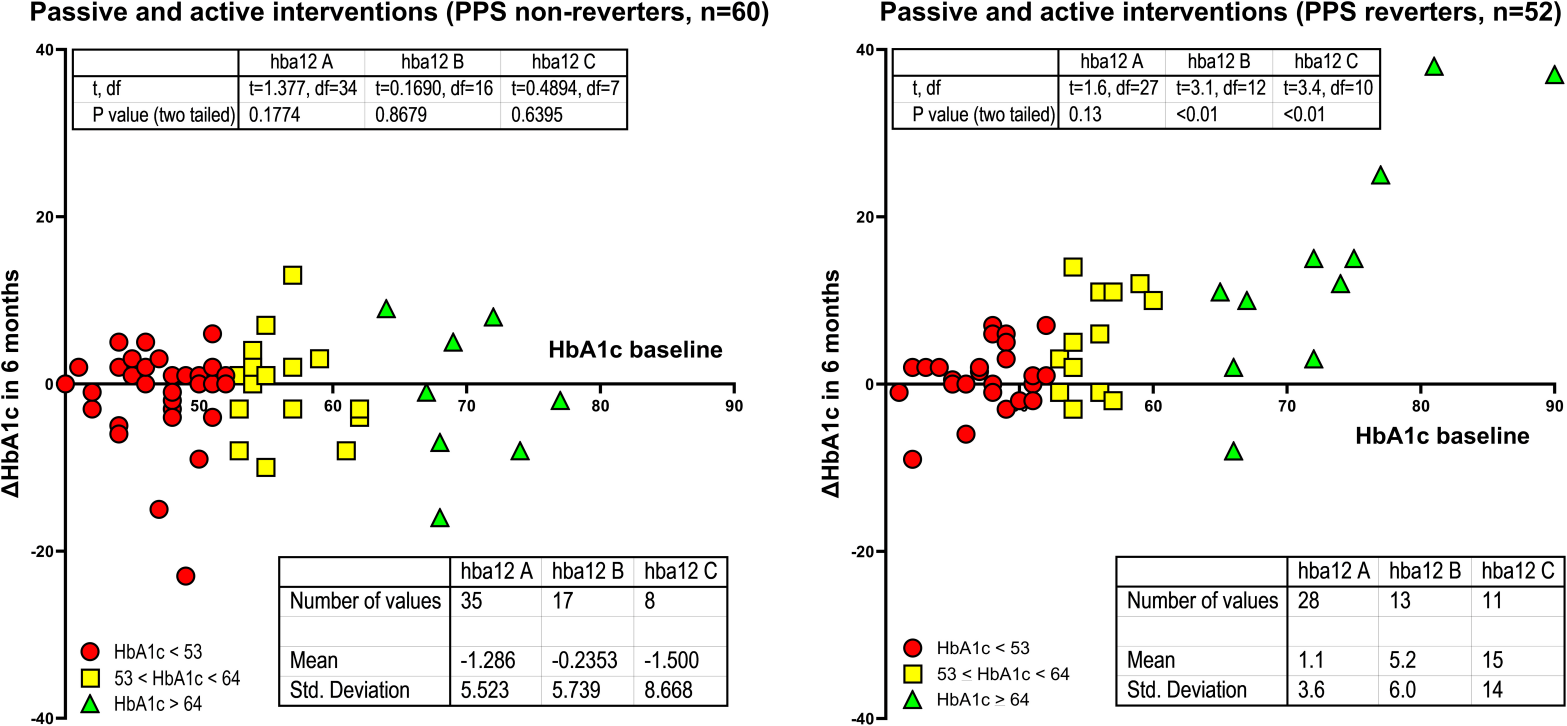
Stratification of baseline HbA1c values into three groups. PPS reverter and PPS non-reverter analyses of HbA1c changes in units of mmol/mol. Differences in mean HbAc1 as change after 6 months of intervention in units of mmol/mol for three levels of baseline HbA1c values. Reverters and non-reverters represented by different colors as shown. Regarding HbA1c change depicted on the y-axis, a positive value reflects a reduction in HbA1c.

**Figure 5:**
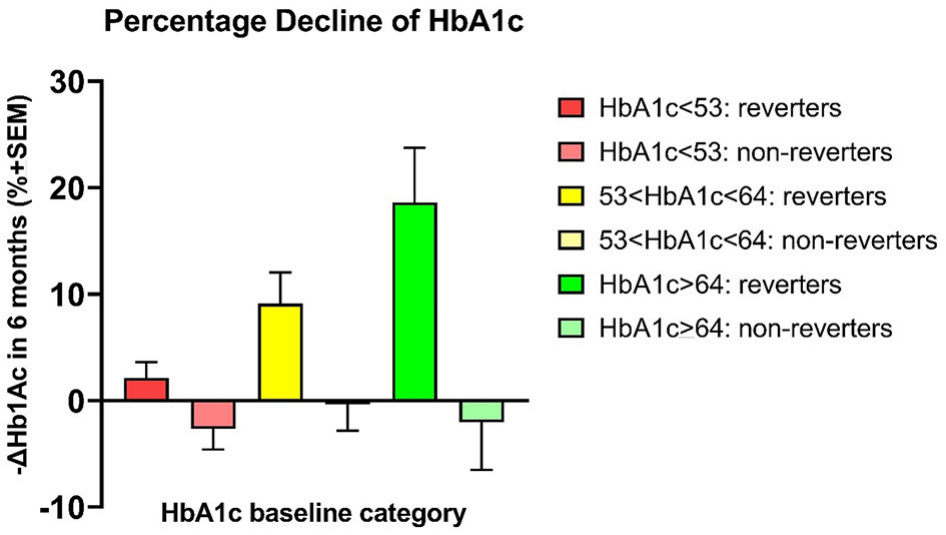
Percentage declines of HbA1c. PPS reverters vs. PPS non-reverters by analysis of percentage HbA1c changes. Differences in mean HbAc1 (mmol/mol before minus after 6 months of intervention) for three sets of patients with a predefined incremental increase in baseline HbA1c between sets.

Stratification according to baseline HbA1c values included members of Group A (HbA1c < 53 mmol/mol), Group B (53 mmol/mol ≤ HbA1c < 64 mmol/mol), and Group C (HbA1c ≥ 64 mmol/mol). For PPS non-reverters, stratification revealed unchanged HbA1c averages in all three groups (P for trend = 0.869) as listed in Table 2 and shown in Figure 4. In contrast, for PPS reverters, stratification revealed a significant trend toward greater reduction of HbA1c in Group A through to Group C (*P* for trend < 0.0001). Among participants with baseline HbA1c ≥ 64 mmol/mol, the reduction of HbA1c averaged 22% (*P* = 0.01) (Cohen Effect 14.9) for PPS reverters compared to PPS non-reverters, as listed in Table 2 and shown in Figures 4, 5. For groups A and B, the corresponding reductions averaged 5 and 9.6%, respectively, as shown in Figure 5.

**Table 2:** Homeostasis effect on HbA1c measured at baseline and after 6 months of intervention, Group of PPS-reverters compared to group of non-reverters, and for three groups of participants based on baseline HbA1c; Group A) HbA1c < 53 mmol/mol; Group B) 53 mmol/mol ≤ HbA1c < 64 mmol/mol; Group C) HbA1c ≥ 64 mmol/mol).

**HbA1c: [mean (SD)] mmol/mol**	***n***	**Baseline**	**Post treatment**	**Between group *P*-value (post-treatment)**	**Cohen's effect size**
**Group A: Baseline HbA1c**<**53 mmol/mol**					
PPS reverters	28	47.4 (3.3)	46.3 (4.2)	0.1	0.6
PPS non-reverters	35	47.3 (3.3)	48.6 (6.5)		
**Group B: 64 mmol/mol** > **HbA1c** ≥**53 mmol/mol**					
PPS reverters	13	55.6 (2.2)	50.5 (5.4)	0.016	3.0
PPS non-reverters	17	56.2 (3.1)	56.4 (6.9)		
**Group C: HbA1c** ≥**64 mmol/mol**					
PPS reverters	11	73.2 (7.6)	58.6 (8.6)	0.01	14.6
PPS non-reverters	8	69.9 (4.2)	71.4 (10.2)		

For explanation of Cohen's effect size see Statistics. The data of this table are shown in Figure 3 as well.

## Discussion

We commonly ascribe the concept of homeostasis to Hippokrates who allegedly alluded to “*The ability of living beings to maintain their own stability as vis mediatrix, that is the existence of agencies ready to operate correcting when the normal state of the organism is unset”* (Cannon, 1926). Claude Bernard introduced a more precise concept of homeostasis, according to which the internal environment of living organisms is more than a vehicle for the delivery of nourishment to cells as “the fixity of the “milieu interieur” which is the condition of free and independent life” (Bernard, 1878-1879). W. B. Cannon suggested the term “homeostasis” for the “coordinated physiological processes which maintain most of the steady states in the organism” (Cannon, 1926) that serve as key functions of the autonomic nervous system after Hans (Janos) Selye's claim of an association with stress (Selye, 1971).

### Main findings

The study showed that the higher the baseline of HbA1c observed in T2DM, the greater the reduction of HbA1c discovered after the 6 months of study, but only in individuals demonstrating a concomitant reduction in sensitivity to painful pressure at the sternum. We conclude that the return to homeostasis of glucose levels in the circulation was associated with concomitant reductions of the PPS measure. An underlying but still unknown mechanism evidently linked the reduction of the PPS measure to HbA1c normalization, as demonstrated by the comparison of reverters to non-reverters. We obtained the proof of concept from the analysis of the group of individuals only receiving conventional anti-diabetic therapy, and subsequently from the analysis of the group of individuals receiving a non-pharmacological treatment in addition to the conventional therapy. We apply this therapy to reduce PPS by a combination of daily home measurements of PPS followed by reflection on daily life practices, daily cutaneous sensory nerve stimulation, and ongoing professional surveillance that leads to proactive contact with the participant if measurements are missing or deviating. The HbA1c values declined by 22% among reverters compared to non-reverters of the stratification group C of participants with baseline HbA1c values greater than 64 mmol/mol, matched by a huge Cohen's effect size of 14.6. The comprehensive non-pharmacological intervention generally used to treat diabetes with education and counseling among other steps leads to reduction of HbA1c, especially for the individuals with the highest baseline HbA1c values. The present study adds to the understanding of these effects by showing that we can expect a beneficial effect only when PPS values undergo a simultaneous reduction. This is an important novel observation. The finding suggests that reversal of ANS dysfunction (ANSD), allegedly reflected in the reduction of elevated PPS values relates to restored glucose homeostasis evidenced by normalization of HbA1c. This reduction in HbA1c matches the results of contemporary pharmaceutical treatment in T2DM.

Saito et al. (2015) previously established a link between ANSD and insulin resistance, raising the possibility that insulin resistance above a certain level is associated with ANSD as cause or effect (Guemes and Georgiou, 2018; Lundqvist et al., 2019; Spallone, 2019) or mutual reinforcement (Lundqvist et al., 2019). If so, reduction of HbA1c in the present study may be the result of lowered insulin resistance (Park et al., 2015). Although we have no direct measure of insulin resistance in the present study (Faber et al., 2021), a previous examination of the intervention in healthy office workers with elevated PPS values did reveal a trend toward an association between reduction of elevated PPS measures and reduction of HOMA-IR (Ballegaard et al., 2014).

The results of the present study in combination with findings in healthy individuals suggest that elevated PPS measures may reflect a disruption of plasma glucose homeostasis, potentially reflecting increased insulin resistance. If so, it is likely that non-pharmacological intervention into the mechanism underlying elevated PPS measures may revert a disrupted glucose homeostasis by lowering the degree of ANSD.

### Contemporary strategic challenges of type 2 diabetes

Present approaches to treatment of T2DM include lifestyle adjustments, and pharmaceutical treatment aimed at lowering the elevated HbA1c levels, as well as risk factor elimination. However, no therapy addresses an aspect of ANSD that may be the key to the understanding of T2DM and its complications (Guemes and Georgiou, 2018; Arrigoni et al., 2019; Lundqvist et al., 2019; Spallone, 2019). We found that reversion of elevated PPS measures has links to improved glucose metabolism. The present study supports this conceptual advance by specifically testing the association between elevated PPS values and disrupted glucose homeostasis, the latter restored by the procedure that lowered the elevated PPS values. We consider this association useful in the context of the current absence of other measures of central autonomic function and approaches that reverse reduced homeostatic functions.

### Clinical perspectives

We accept the general claim that homeostasis is subject to control by the ANS, but the underlying mechanisms vary according to target. However, we hold that homeostasis generally is essential to survival (Goldstein, 2019). The agreement raises the question of the strength of the evidence, to which the answers vary. The findings of three independent consecutive RCT suggest that PPS measures relate to the ANS function system (Faber et al., 2021). Recently we suggested that PPS measures may relate to the orexin cell system of the lateral hypothalamus and as such reflects a central cerebral regulation of ANS (Faber et al., 2021). In consecutive and independent reports of RCT, reduction of elevated PPS values reflected lowering of important health risk factors, assumed to be under the control of ANS in the brain and to be of mutual importance to individuals with IHD and T2DM. The risk factors include depression (Faber et al., 2021), heart rate, blood pressure, work of the heart, serum cholesterol (Ballegaard et al., 2014), blood pressure response to tilt-table test (Faber et al., 2021), and glycated hemoglobin (Faber et al., 2021). Elevated PPS measures are prevalent among approximately 60% of individuals with T2DM, whether controlled in general practice or in specialized hospital clinics (Faber et al., 2021).

### Strengths and limitations

#### Strengths

The claim of the null hypothesis that reduction of elevated PPS measures would be expected to be unrelated to an association between normalization of HbA1c and baseline HbA1c values after 6 months of intervention has not previously been tested in healthy volunteers or in individuals with T2DM. The conventional intervention group was not a genuine control group as the subjects of that group received no placebo treatment but did consist of individuals informed by the researchers of the elevated PPS measure that represent a signal of a potentially unhealthy state. The information may have inspired attempts of self-regulation by the members of the conventional intervention group that may explain how approximately 25% of the recipients of conventional intervention obtained a predefined minimum reduction of the elevated PPS measure. However, the information more strongly suggests that reductions of PPS are associated with decline of HbA1c measures that are unrelated to specific causes of lowered PPS values. We regard the possible risk of type 1 error as small. We conducted two independent tests with the same results, i.e., that PPS reverters experienced a homeostatic normalization that was independent of randomization to conventional or conventional-plus-experimental intervention. Nonetheless, the likelihood of PPS reversion of members of the active experimental intervention group exceeded the likelihood of reversion of members of the conventional group by a factor of 5, although the significance of this result is not evident.

#### Limitations

The intervention included repeated PPS measures at home, together with cognitive reflection, sensory nerve stimulation, and professional surveillance, but the design did not distinguish between individual effects of these components. We know that cognitive reflection on home measurements of HbA1c can improve measures of glucose metabolism (Wada et al., 2020), as can continuous professional surveillance by means of telemedicine (Faruque et al., 2017). In addition, we know that cutaneous sensory nerve stimulation has effects on central ANS mechanisms (Ballegaard et al., 1993). Although the methods of sensory stimulation are different, it is possible that the same afferent signals reach the central ANS for different approaches to stimulation of nervous and brain tissue. Neuronal stimulations include vagal or sacral nerve stimulations (Kupers et al., 2006; Lundby et al., 2011; Landau et al., 2015), spinal cord stimulation (Tesfaye et al., 2010; Richner et al., 2019), and non-noxious cutaneous sensory nerve stimulation (Uvnäs-Moberg et al., 2014). Also, sensory nerve stimulation is known to reduce pain and restore normal autonomic function (Uvnäs-Moberg et al., 2014), to enhance cardiovascular homeostasis (Ballegaard et al., 1993), to reduce diabetic neuropathy (Tesfaye et al., 2010), and to reduce angina pectoris (Richner et al., 2019), in relation to the well-known autonomic reflex regulation of pain sensation (Arendt-Nielsen and Yarnitsky, 2009; Yam et al., 2018). It is a possible limitation that we did not conduct specific neuropsychological assessments at the time of testing. Instead, we used questionnaires of same-day self-reported mood and emotional, social, and mental functions (Faber et al., 2021), as listed in Table 1. This revealed no significant between-group differences.

## Conclusion

In consecutive analyses of two different populations of individuals with T2DM, we demonstrated that the higher the baseline HbA1c, the greater the reduction of HbA1c but only in individuals with a concomitant reduction of sensitivity to PPS, suggesting a homeostatic effect of the autonomic nervous system on glucose metabolism. As such, ANS function, measured as PPS, is an objective measure of HbA1c homeostasis. This observation may be of great clinical importance.

## Data availability statement

The original contributions presented in the study are included in the article/supplementary material, further inquiries can be directed to JF.

## Ethics statement

The study was approved by the Ethical Committee of the Capital Region, Denmark (ID) (identifier: H 17034836). The patients/participants provided their written informed consent to participate in this study.

## Author contributions

JF is a major contributor to the study. SB is the inventor of the device and intervention used and had a major role in design of the study, education of the patients in the active group, discussion of the manuscript, and created the concept of an association between an elevated measure of pressure pain sensitivity at the chest bone and disrupted glucose homeostasis and between restoration of glucose homeostasis and reduction of this measure. EE has been the blinded diabetes specialist of the study and participated in interpretation of the results and preparation of the manuscript. AG had the major role in the interpretation of the results and preparation of the manuscript and conducted statistical analyses. FG, SH, NØ, and BK had roles in interpretation of the results and preparation of the manuscript. All authors contributed to the manuscript and approved the submitted version.
